# Short-term heat acclimation protocols for an aging population: Systematic review

**DOI:** 10.1371/journal.pone.0282038

**Published:** 2023-03-02

**Authors:** Edward Cole, Kate J. Donnan, Andrew J. Simpson, Andrew T. Garrett

**Affiliations:** Faculty of Health Science, School of Sport, Exercise and Rehabilitation Sciences, University of Hull, Hull, Yorkshire, United Kingdom; University of Hertfordshire, UNITED KINGDOM

## Abstract

**Introduction:**

Elderly and sedentary individuals are particularly vulnerable to heat related illness. Short-term heat acclimation (STHA) can decrease both the physical and mental stress imposed on individuals performing tasks in the heat. However, the feasibility and efficacy of STHA protocols in an older population remains unclear despite this population being particularly vulnerable to heat illness. The aim of this systematic review was to investigate the feasibility and efficacy of STHA protocols (≤twelve days, ≥four days) undertaken by participants over fifty years of age.

**Methods:**

Academic Search Premier, CINAHL Complete, MEDLINE, APA PsycInfo, and SPORTDiscus were searched for peer reviewed articles. The search terms were; (heat* or therm*) N3 (adapt* or acclimati*) AND old* or elder* or senior* or geriatric* or aging or ageing. Only studies using primary empirical data and which included participants ≥50 years of age were eligible. Extracted data includes participant demographics (sample size, gender, age, height, weight, BMI and $\dot{\text{V}}{\text{O}}_{2\text{max}}$), acclimation protocol details (acclimation activity, frequency, duration and outcome measures taken) and feasibility and efficacy outcomes.

**Results:**

Twelve eligible studies were included in the systematic review. A total of 179 participants took part in experimentation, 96 of which were over 50 years old. Age ranged from 50 to 76. All twelve of the studies involved exercise on a cycle ergometer. Ten out of twelve protocols used a percentage of $\dot{\text{V}}{\text{O}}_{2\text{max}}$ or $\dot{\text{V}}{\text{O}}_{2\text{peak}}$ to determine the target workload, which ranged from 30% to 70%. One study-controlled workload at 6METs and one implemented an incremental cycling protocol until T_re_ was reached +0.9°C. Ten studies used an environmental chamber. One study compared hot water immersion (HWI) to an environmental chamber while the remaining study used a hot water perfused suit. Eight studies reported a decrease in core temperature following STHA. Five studies demonstrated post-exercise changes in sweat rates and four studies showed decreases in mean skin temperature. The differences reported in physiological markers suggest that STHA is viable in an older population.

**Conclusion:**

There remains limited data on STHA in the elderly. However, the twelve studies examined suggest that STHA is feasible and efficacious in elderly individuals and may provide preventative protection to heat exposures. Current STHA protocols require specialised equipment and do not cater for individuals unable to exercise. Passive HWI may provide a pragmatic and affordable solution, however further information in this area is required.

## Introduction

Performing tasks in a hot environment causes physiological stress and impaired performance in non-acclimated individuals [1]. Heat acclimation (HA) can decrease both the physical and mental stress’ imposed on individuals performing tasks in the heat. This is particularly important for older populations, as they are at an increased risk of heat related illness [2,3]. This is due to a deterioration the ability to dissipate heat, specifically an inhibited sweat response [4,5]. This deterioration is caused by reduced functioning of the sweat glands, which increase and maintain sweat rates. In older males and females, the response from the eccrine glands is delayed, causing a higher core temperature threshold needed to trigger the onset of sweating which results in reduced effectiveness [4]. This ultimately results in elderly individuals experiencing higher core temperatures and greater heat strain in response to hot environments [4].

Older populations have a greater mortality risk during heat waves, evidenced by an exponential increase in mortality in people over the age of fifty, with most medical complications and deaths during heatwaves originating from increased cardiovascular strain [2,3]. Older (over 50) populations are underrepresented in HA literature [6]. Additionally, consideration to individuals over 50 who remain in occupations such as the military and firefighters is needed as they are often exposed to strenuous work in hot conditions where they are at increased risk of developing heat related illness [7,8]. In a time where hotter weather is becoming more frequent, longer lasting and more dangerous, it is important to explore different methods of heat adaptation, such as hot water immersion, which may be more suitable for older and underrepresented populations [9,10].

Heat acclimation protocols vary in duration, both in terms of session length, number of days of exposure and whether they are consecutive or non-consecutive, the environmental temperature and humidity used, or whether a passive or exercise-based protocol are used, which means HA can be tailored to the needs of a specific population. Given the time constraints of modern weather prediction [11], short-term heat acclimation (STHA) seems the most viable choice as a pre-emptive heatwave protection method, compared to medium-term and long-term heat acclimation. While both medium-term and long-term heat acclimation provide more complete adaptations, STHA (typically four to seven days) is sufficient for up to 80% of the adaptations occur [12]. As modern weather prediction techniques can forecast up to twelve days in advance [11], STHA of ≥4 days [12], could be feasible as a pro-active protection method. Daanen and Herweijer [13] found that three-days of HA provided no physiological benefits in young or elderly females suggesting that an effective STHA protocol would have to include more than three consecutive days of HA. Whilst based on just 15 participants and exploring just three days of HA, this raises questions as to whether there may be sex differences in the effectiveness of HA. A recent paper by Mee *et al*. [14] found that females need longer daily exposures or more days of HA compared to males. While five days HA has been shown to be effective in females [10] four days has yet to be trialled. There may also be differences in the effectiveness for females who have undergone menopause, but limited thermoregulatory research has investigated the effectiveness of STHA in young and older females. Sex differences in the effectiveness of HA therefore needs further exploration.

Controlled hyperthermia is one technique of achieving HA that involves repeated sessions of submaximal work in an environmental chamber to increase the participant’s core temperature above 38.5ºC. The time that a participant spends above 38.5ºC is important for developing physiological adaptations [15]. Consecutive bouts of controlled hyperthermia have the potential to cause adaptive responses that can reduce thermal strain and improve performance. Some examples of these responses are reduced resting and exercising core temperature, reduced heart rate, caused by increased stroke volume and arterial blood pressure that results in increased cardiovascular stability [16,17]. In addition, increased sweat rate enhances thermal comfort that in-turn leads to greater exercise capacity [18].

Passive heating can induce numerous health benefits, similar to those induced by sustained exercise (e.g., improved vascular health, glycaemic control) [19]. As exercise is not feasible for all individuals, passive heating my offer an alternative method of eliciting these health benefits. Another psychological adaptation from HA is that it can attenuate increased sensations of fatigue during exercise-heat stress [20]. The physiological effects of HA greatly impacts the cardiovascular system, these adaptations gained from HA could reduce the physiological strain of daily living activities of an older population and in its most extreme cases may provide a protective effect against heat related illness during heatwaves [3].

Heatwaves are becoming more extreme and more frequent across the globe [21], thus there is increased incentive for vulnerable individuals such as older populations to adopt strategies that reduce physiological stress during times of increased environmental temperatures. There are numerous strategies devised to mitigate the immediate effects of heat waves, such as fans (electric and hand powered), wet T-shirts, pre-cooling, avoiding strenuous activity and avoiding long periods in direct sunlight, staying indoors as much as possible, air conditioning, wearing loose-fitting clothing, wearing a wide brimmed hat, staying hydrated, seeking shade, applying sun cream and avoiding alcohol and caffeine [4]. However, STHA as a pre-emptive heatwave preparation strategy is yet to be fully explored.

Millyard *et al*. [4] identified that active HA is currently an impractical way of protecting vulnerable people against heat waves due to the need for specialised equipment. For example, core temperature measures that are essential for safety reasons and the use of an environmental chamber, which can be expensive and hard to access. A more practical and affordable approach to STHA such as hot water immersion (HWI) could provide a solution. A five-day HWI immersion protocol has been shown to have the same effect and confer the similar physiological and psychological adaptations in an older population when compared to an active HA protocol [10]. Further, the adaptations gained from HWI have been reported to remain for up to two weeks [22]. This area of research could provide an exciting new avenue for HA.

The aim of this systematic review was to determine the feasibility and efficacy of short-term heat acclimation (STHA) protocols undertaken by healthy participants over fifty years of age.

## Materials and methods

### Protocol

This review was completed in accordance with the relevant items in the preferred reporting items for systematic reviews and meta-analyses (PRISMA) [23].

### Search strategy

An initial comprehensive search was performed on 10^th^ September 2022. The search terms were as follows; (heat* or therm*) N3 (adapt* or acclimati*) AND old* or elder* or senior* or geriatric* or aging or ageing. The final search was done on 18^th^ October 2022, no additional papers were found.

The search was conducted using EBSCOhost Research Databases using the advanced search function that including the following data bases: Academic Search Premier; CINAHL Complete; MEDLINE; APA PsycInfo; SPORTDiscus with Full Text. Only published peer reviewed articles available in English were included. Only studies using primary empirical data were eligible.

### Data extraction and analysis

The relevant data was then extracted from the articles and entered into two tables. Table 1 containing participant demographics; sample size, gender, age, height, weight, BMI and $\dot{\text{V}}{\text{O}}_{2\text{max}}$ (ml/kg/min). Table 2 containing; acclimation protocol details including, pre to post protocol, activity, frequency, duration and outcome measures taken.

**Table 1:** Participant data.

Study (n = 12)	Sample size	Gender	Age	Height (cm)	Weight (kg)	BMI	$\dot{\text{V}}{\text{O}}_{2\text{max}}$ (ml/kg/min)
Anderson et al 1987 [24]	n = 16 8 older 8 younger	Female	56±4, 25±4	167±8, 165±11	55.6±11, 59.3±11	Not reported	34.4±5, 36.9±6
Armstrong et al 1993 [25]	n = 12 6 older, 6 younger	Male	61±1, 26±2	173±2, 177±2	71±1, 74±2	Not reported	43.8±2, 45.9 ±1
Best et al 2014 [26]	n = 14 7 older 7 younger	Unspecified	50–63, 19–32	“Matched for height, body mass and training volume”			
Fuji et al 2021 [27]	n = 8 8 older	Male	59±9	189±0.1	75.1±8	Not reported	43.3±10
Gerrett et al 2021 [28]	n = 10 10 older	Mixed 8M 2F	67±2	165.9±7	60.8±9	Not reported	32.6±5
Inoue et al 1999 [29]	n = 14 5 untrained older 4 trained older 5 trained younger	Male	67±3, 63±3, 23±1	178±2, 176±3, 177±2	73±3, 71.1±2, 73.9±4	Not reported	30.1±1, 47.5±4, 47.1±3
Kenney et al 1988 [30]	n = 16 8 older, 8 younger	Female	56±1, 25±1	167±3, 165±3	55.6±4, 59.3±4	Not reported	34.4±2, 36.9±2
Macartney et al 2021 [31]	n = 18 8 with type 2 Diabetes 10 without	Male	50–70 50–70	“Similarly matched”			
Notley et al 2019 [32]	n = 18 8 with type 2 Diabetes 10 without	Male	58±5, 61±6	176±0, 174±0	85±13, 77±5	27.6±4 25.6±2	32±7 37±5
Takamata et al 1999 [33]	n = 15 9 older, 6 younger	Male	70±3, 25±3	161±6, 168±4	61.7±8, 64.3±4	23.7±1, 22.7±1	31±2, 57±3
Waldock et al 2021 [10]	n = 26 8 older 7 older HWI 11 younger	Mixed 7M 1F 3M 4F 8M 3F	68±3, 73±3, 22±2	175±9, 162±7, 175 ±6	74.1±11, 71±20, 74±14	24.2±3, 27.1±6, 23.9±4	Not reported
Zappe et al 1996 [34]	n = 12 6 older, 6 younger	Male	67±1, 24±2	Not reported	76.6±4, 87.1±9	Not reported	38±3, 45±4

HWI = Hot water immersion, $\dot{\text{V}}{\text{O}}_{2\text{max}}$ = Maximum oxygen uptake.

**Table 2:** Heat acclimation activity, frequency and duration of heat acclimation and outcome measures.

Study (n = 12)	Pre to post protocol	HA Activity	Frequency and duration of HA	Outcome measures in older groups following HA (significant directions of change shown ↑↓)
Anderson et al 1987 [24]	Treadmill 35–40% $\dot{\text{V}}{\text{O}}_{2\text{max}}$ for 2 hours or until test termination due to participant safety, dehydration	Treadmill or cycling 30% $\dot{\text{V}}{\text{O}}_{2\text{max}}$	Five to ten exercise sessions, 25°C wet bulb, to volitional exhaustion or 39°C T_re_. until acclimation was achieved as defined by similar HR and T_re_ between consecutive sessions and a levelling off of HR and T_re_ within the last 30 minutes, *Ad libitum* Hydration,	%HR_max_, SR_b_ (↓), SR_l_ (↓), M_sw_, HASG, sweat gland output (↓) T_sk_, T_re_ (↑), T_b_ (↑), rate of heat storage (↑)
Armstrong et al 1993 [25]	30 minutes baseline, 28°C 28%RH, 60 minutes thermal transient, 28°C to 46°C 11%RH, 30 minutes 46°C	Treadmill or cycle at 40% $\dot{\text{V}}{\text{O}}_{2\text{max}}$	Nine-day passive HA within ten-days in, 28°C wet bulb and 27% RH, 1.5/2-hours per day.	FBF (↓), HR (↓) SR_ch_ (↓) T_b_ (↓), T_sk_ (↓), T_re_ (↓)
Best et al 2014 [26]	Two 60-minute HSTs pre and post HA, one in thermoneutral (20°C, 40%RH) and on in hot conditions (35°C, 40%RH).	Cycling 70% $\dot{\text{V}}{\text{O}}_{2\text{max}}$	Six-days, HA 60 minutes in 35°C and 40%RH.	%HR_max_ (↓), CVC, MAP (↓), PV, Q, HR M_sw_ T_b_ (↓), T_re_ (↓), T_sk_ (↓), _$\dot{\text{V}}{\text{O}}_{2\text{peak}}$,_
Fuji et al 2021 [27]	Calorimetry, Semi- recumbent cycling at 150W, 200W, 250W, (15 minutes rest, 3 x 30 minutes, 15 minutes rest at 40°C 20%RH) Micro dialysis, two habituation sessions, Semi-recumbent cycling 400W ≈40% $\dot{\text{V}}{\text{O}}_{2\text{peak}}$ (75 minutes at 25°C 20%RH up to 35°C 20%RH for another 75 minutes, 60 minutes of exercise component followed by 30 minutes recovery).	Semi- recumbent cycling 50% $\dot{\text{V}}{\text{O}}_{2\text{peak}}$	Seven-days of euhydration HA in 40°C 20% RH for 90 minutes.	HR (↓), MAP NOS and COX inhibition. Evaporative heat loss (↓) T_sk_ (↓), ΔT_re_, T_re_ (↓)
Gerrett et al 2021 [28]	Water perfused suit 38°C and legs submerged in a 42°C water bath for 60 minutes in 25°C 50% RH ambient temperature.	Cycling, started at 75W then increased by 10-20W every 5 minutes afterwards.	Nine-non-consecutive days-controlled hyperthermia euhydration HA, 35°C and 45% RH completed within 14-days. HA increase T_re_ by 0.9°C maintain for 60 minutes.	HR, BV, CVC (↑), MAP, PV (↑), SkBF, Estimated $\dot{\text{V}}{\text{O}}_{2\text{max}}$ (↑), GSL, HASG, NaCl, SR (↑), SR onset temperature (↓), sweat gland ion reabsorption (↑ chest only) T_b_, T_sk_, T_re_ (↓) TC, TS (↓), Wetness and thirst sensation, RPE Aldosterone
Inoue et al 1999 [29]	Methylcholine-induced sweating test	Cycling, 35% $\dot{\text{V}}{\text{O}}_{2\text{max}}$	Eight-days x 90 minutes, 43°C 30%RH with two rest days between day 4 and 5 of HA.	$\dot{\text{V}}{\text{O}}_{2\text{peak}}$ (days 1, 3, 5, 7 and 8) SR, total body SW (↑), M_sw_ (days 1,3,5,7 and 8), sodium concentration (days 1 and 8), sweat gland output (↑). T_re_ (↓), T_sk_ TC (↓)
Kenney et al 1988 [30]	Treadmill 35–40% $\dot{\text{V}}{\text{O}}_{2\text{max}}$ for 2 hours or until test termination due to participant safety, dehydration	Treadmill or cycling 30% $\dot{\text{V}}{\text{O}}_{2\text{max}}$	five to twelve sessions (2 hours max each), in alternating humid dry and wet humid environments, until acclimation status was achieved as defined by similar HR and T_re_ between consecutive sessions and a levelling off of HR and T_re_ within the last 30 minutes, *Ad libitum* Hydration.	$\dot{\text{V}}{\text{O}}_{2\text{max}}$, PV (only in the Wet humid conditions ↓), Body surface area, M_sw_, body heat storage (only in humid dry conditions ↑), T_re_ (↑), T_sk_
Macartney et al 2021 [31]	HST, 3 x 30 minute bouts, cycling 150W, 200W and 250W, 40°C and 15%RH.	HA, 50% $\dot{\text{V}}{\text{O}}_{2}$ Peak	Seven-days, 90 minutes, 40°C and 15%RH	Rest HR (↓), Exercising HR (↓), HRRec, T_re_ Detrended fluctuation analysis alpha-1, detrended fluctuation analysis alpha-2.
Notley et al 2019 [32]	HST, 3 x 30 minute bouts, cycling 150W, 200W and 250W, 40°C and 15%RH.	HA, 50% $\dot{\text{V}}{\text{O}}_{2}$ Peak	Seven-days, 90 minutes, 40°C and 15%RH	HRR (↓) Whole body heat loss (↓), dry heat loss, evaporative heat loss(↓) T_b_ (↓)
Takamata et al 1999 [33]	HST, 4 x 20 minute bouts, Semi recumbent cycling, 10 minutes recovery, 36°C 40% RH.	Semi recumbent cycling, 40% $\dot{\text{V}}{\text{O}}_{2}$ peak,	HA, Six-day exercise dehydration, 4 x 20-minute bouts to maintain HR, separated by 10 minutes resting, 36°C 40% RH.	PV, BV, P_osmol_ (↑), sodium concentration, Thirst rating, fluid intake Plasma arginine vasopressin concentration, plasma renin activity, aldosterone concentration, renal function.
Waldock et al 2021 [35]	HST, 30 minutes rest, 30 minutes semi- recumbent cycling at 6 METs/3.5W/kg^-1^ in 35°C 50% RH.	Passive exposure, Graded cycling exercise test to elicit 6METs and 3.5W/kg^-1^ 6MWT, ±0.2KM/h^-1^ “Simulated daily living protocol” 30 minutes rest, 30 minutes recumbent cycling at 6 METs/3.5W/kg^-1^ Exercise HA conditions (young and old), recumbent cycling, (men 1.5W/ kg^-1^ women 1.2W/ kg^-1^) to achieve 38.5°C or +1.5°C HWI group (exclusively old) identical relative exercise, not an objective to get to T_re_ 38.5°C, resting in blankets.	30 minutes passive exposure, all tests were completed in 35°C 50% RH. Five-days HA, Exercise conditions, 35°C 50%RH, if core temperature was >36.5°C within 60mins, the trail was maintained for a further 60mins, if the exercise level couldn’t be maintained then it was reduced to continue exercise. Five-days HA, HWI condition, 30 minutes exercising, 23°C and 60%RH, 30 minutes HWI, 40°C up to sternum, 30 minutes rest under blankets.	HR, %HR_max_, SBP (↓), DBP, MAP (↓) WBSR Thermal impulse, duration T_re_ > 38.5°C, duration T_re_ > +1.5°C, T_re_ (↓), T_re_ AUC 38.5°C, T_sk_ rest, T_sk_ peak (↓) RPE (↓), TC, TS (↓) Exercise duration
Zappe et al 1996 [34]		Semi- recumbent cycling, 50% $\dot{\text{V}}{\text{O}}_{2\text{max}}$	Four-days HA, 30°C wet bulb 60% RH, 60 minutes resting, 90 minutes exercising, 150 minutes resting.	PV, U_na_V, U_osm_, UV, ERPF, GFR, FR_d_, FR_p_, concentrations of K^+^, Na^+^ (↓), [TP], urea and glucose, Fluid intake, urine output, body weight, PRA Aldosterone, ADH (↑).

6MWT = 6-minute walk test, ADH = antidiuretic hormone., BP = Blood Pressure, BV = Blood Volume, COX = Cyclo-oxygenase, CVC = Cutaneous Vascular Conductance, DBP = Diastolic blood pressure, ERPF = Effective Renal Plasma Flow, FBF = Forearm Blood Flow, FRd and FRp = Fractional Distal and Proximal Tubule Reabsorption, GFR = Glomerular Filtration Rate, GSL = Gross Sweat Loss, HA = Heat Acclimation, HASG = Heat Activated Sweat glands, HR = Heart Rate, HRR = Heart Rate Reserve, HRRec = Heart rate recovery, HST = Heat Stress test, HWI = Hot Water Immersion, MAP = Mean Arterial Pressure, METs = Metabolic Equivalents, M_sw_ = Mean Sweat Rate, NOS = Nitric Oxide Synthase, PRA = Plasma renin activity, PV = Plasma Volume, $\dot{\text{Q}}$ = Cardiac Output, RH = Relative Humidity, RPE = Rate of Perceived Exhaustion, SBP = systolic blood pressure, SR_ch_ = Sweat Rate (chest), SR_l_ = Sweat Rate (Local), SR_b_ = Sweat Rate (whole body), TC = Thermal Comfort, TS = Thermal sensation, T_b_ = Body Temperature, Tre = Rectal Temperature, Tsk = Skin temperature, UV = Urine Flow Rate, UnaV = Urine Sodium excretion, Uosm = Urine Osmolality, $\dot{\text{V}}{\text{O}}_{2\text{max}}$ = Maximum oxygen uptake, $\dot{\text{V}}{\text{O}}_{2\text{peak}}$ = Peak oxygen uptake, WBSR = Whole Body Sweat Rate.

### Inclusion/Exclusion criteria

The intervention was any form of short-term heat acclimation that were equal to or longer than four [13], but no more than twelve HA days, as this is the latest time that a heatwave can be accurately predicted via a weather report [11]. The specific outcome measures being assessed were those conducive to generating heat adaptive responses that can be considered desirable for protection against heat waves either being physiological such as any cardiovascular measures or psychological e.g., reduced perception of fatigue [20]. The final articles were reviewed to assess if any adverse events were reported, this is in the interest of feasibility and safety for future protocols. No restrictions on the timeframe of publication were implemented and only peer-reviewed studies using primary empirical data were eligible.

All included studies were assessed for methodologic quality and risk of bias. The included studies were subject to a modified Downs and Black (1998) checklist to assess the overall quality of the papers and rank them accordingly [36–38]. The modified checklist uses an altered scoring system for item 27 that refers to the power of the study [37–39]. Accordingly, the maximum score for item 27 was reduced from five to one thus the highest possible score for the checklist was twenty-seven. Downs and Black (1998) score ranges were given corresponding quality levels as previously reported [37]: excellent (26–28); good (20–25); fair (15–19); and poor (<14).

## Results

**Fig 1:**
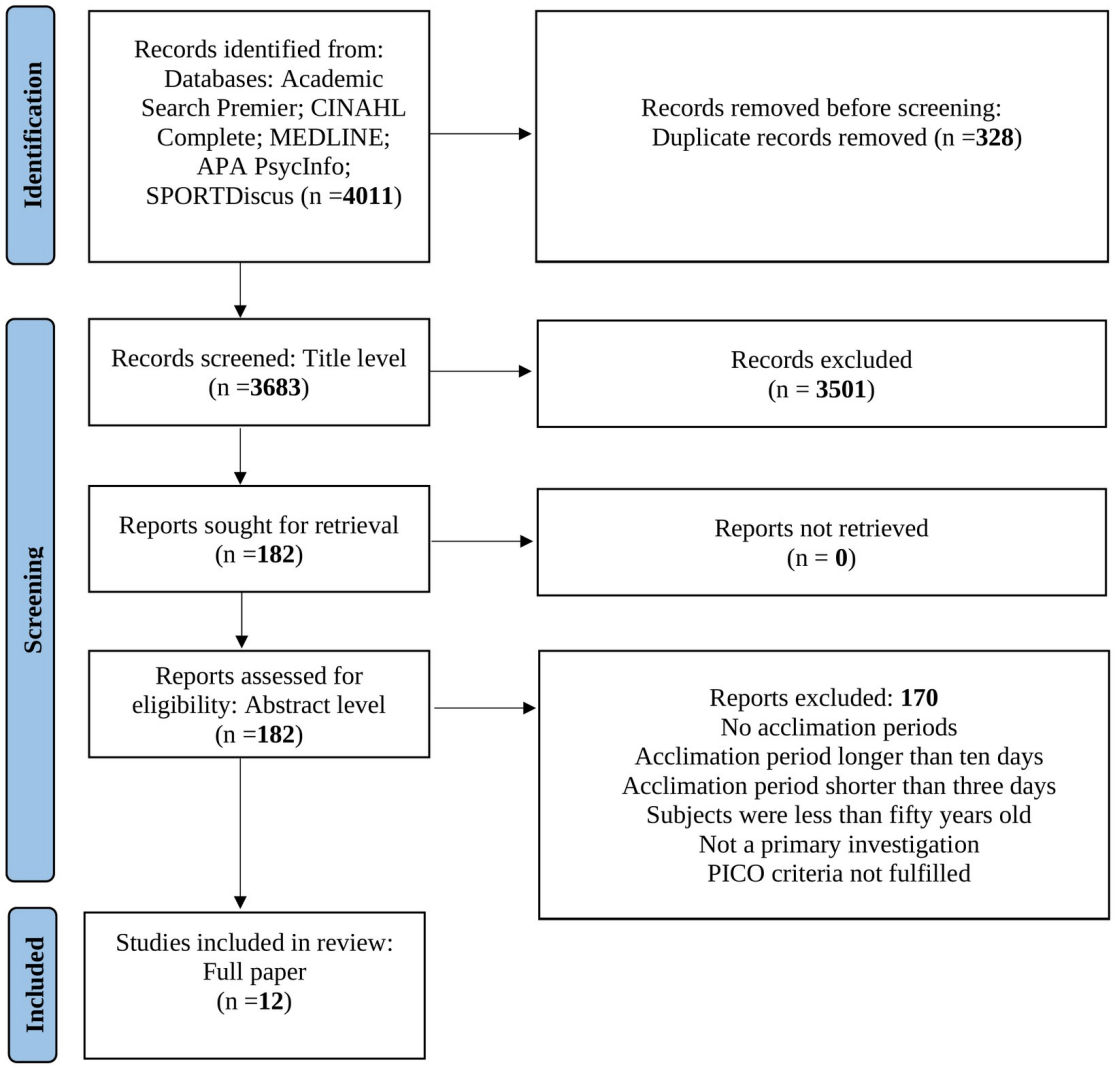
PRISMA flow diagram.

The search returned 4011 studies, of which 328 were duplicates, a further 3501 were deemed not relevant at title level leaving 182 to be assessed at abstract level. A further 170 papers were then excluded for the following reasons; the protocol did not include an acclimation period, the acclimation period was longer than twelve days, the acclimation period was shorter than four-days, the participants were younger than fifty, it was not a primary investigation or the PICO criteria were not fulfilled. Twelve papers were selected for a full text review (Fig 1).

### PRISMA flow diagram

Fig 1 shows the PRISMA flow diagram.

### Article data

Table 1 shows the participant data extracted from the twelve papers selected. The twelve studies had a total of 179 participants (122 older participants) from over four decades. Of the 122 older participants, 23 were older females. However, Kenney and Anderson [30] and Anderson and Kenney [24] utilised the same cohort of participants and this is also the case for Notley *et al*. [32] and Macartney *et al*. [31]. This further reduces the overall number of older volunteers to 96 (15 older females). Age ranged from 50 to 76 in the older groups. Studies were performed in United Kingdom, Canada, United States, Australia and Japan.

Table 2 details the pre to post protocols, HA activity, frequency and duration of HA and outcome measures for all twelve studies. All twelve of the studies involved cycling in some form, four articles specified semi-recumbent cycling, either as part of the HA or during a heat stress test (HST). Each study adopted a different approach to STHA but ten of the twelve studies used a percentage of $\dot{\text{V}}{\text{O}}_{2\text{max}}$ or $\dot{\text{V}}{\text{O}}_{2\text{peak}}$ to generate workload. Waldock *et al*. [10] adopted 6METs and 3.5W/kg^-1^ while Gerrett *et al*. [28] asked their participants to cycle at 75W and then increased the wattage by 10-20W every 5 minutes. The lowest load used was 30% $\dot{\text{V}}{\text{O}}_{2\text{max}}$ [24,30] and the highest was 70% $\dot{\text{V}}{\text{O}}_{2\text{max}}$ [26]—the latter was performed by highly trained cyclists.

Two studies used heated water in some capacity, Gerrett *et al*. [28] utilised a water perfused body suit at 38°C while also submerging the legs in 42°C water for 60 minutes. Waldock *et al*. [10] used HWI as one of the conditions for HA, participants were required to attend five-days HA, where they would undergo 30 minutes exercising in 23°C and 60%RH followed by 30 minutes HWI at 40°C in a water bath up to sternum. The shortest HA protocol was four-days [34] while the longest HA protocol was up to twelve sessions conducted daily until acclimation status had been achieved as defined by similar HR and Tre between consecutive sessions and a levelling off of HR and Tre within the last 30 minutes [30].

Reported outcome variables are shown in Table 2 in addition to the direction of the significant changes observed.

### Modified Downs and Black (1998) [36] scores

Table 3 shows the modified Downs and Black (1998) [36] scores of papers selected from the systematic review. From the quality check, eight papers achieved good status [10,26–32], and the remaining four achieved fair status [24,25,33,34], as per Hooper *et al*. [37]. Scores ranged from eighteen to twenty-two out of twenty-seven suggesting similar quality across studies. All articles performed well in the reporting, external validity, and internal validity (bias). However, the articles did not score highly on internal validity (confounding), with all the articles scoring only two points out of a possible six due to the nature of heat acclimation not allowing for group randomisation where all participants undertook heat acclimation, as well as most articles failing to report when and how they recruited the population. Questions 14 and 15 were scored as a zero for all papers. However, these questions refer specifically to blinding the participants to the interventions and given the nature of HA this is unable to be achieved. The articles had areas that were scored as “unable to determine”. For example, it was impossible to determine the time frame in which the participants were recruited for some of the studies.

**Table 3:** Modified Downs and Black (1998) [36] scores.

Study (n = 12)	Reporting (11)	External Validity (3)	Internal validity: bias (6)	Internal validity: confounding (6)	Power (1)	Total score (27)	Quality score
Anderson et al 1987 [24]	9	3	5	2	0	19	**Fair**
Armstrong et al 1993 [25]	9	2	5	2	0	18	**Fair**
Best et al 2014 [26]	10	3	5	2	0	20	**Good**
Fuji et al 2021 [27]	11	3	5	2	1	22	**Good**
Gerrett et al 2021 [28]	10	3	5	2	1	21	**Good**
Inoue et al 1999 [29]	10	3	5	2	1	20	**Good**
Kenney et al 1988 [30]	10	3	5	2	0	20	**Good**
Macartney et al 2021 [31]	9	2	5	2	0	20	**Good**
Notley et al 2019 [32]	9	2	5	2	0	20	**Good**
Takamata et al 1999 [33]	9	2	5	2	0	18	**Fair**
Waldock et al 2021 [35]	10	3	5	2	1	21	**Good**
Zappe et al 1996 [34]	9	3	5	2	0	19	**Fair**

Modified Downs and Black (1998) [36] scoring [37]; excellent (26–28); good (20–25); fair (15–19); and poor (<14).

## Discussion

The primary aim of this review was to explore the feasibility and efficacy of the use of STHA protocols for an older population. The systematic review identified twelve articles that were eligible for review. Twelve studies using different HA protocols, demonstrated STHA is feasible in an older population. Despite using different methods, all of the studies presented significant changes in physiological outcome measures associated with adaptations brought about by HA. All of the study protocols involved an exercise component. This systematic review suggests that HA is feasible and efficacious in elderly individuals and may provide protection to heat exposures in this population.

Six of the twelve studies reported a decrease in core temperature from HA during exercise [10,25–29], four studies reported decreases in skin temperature during exercise [10,25–27]. Of the six studies that included sweat rates as outcome variables, significant increases in sweat rates were reported in four [25–27,29]. Fujii *et al*. [27] reported a 23% reduction in body heat storage, as a result of improved evaporative heat loss caused by the increased size and efficiency of eccrine sweat glands. This is concurrent to another study showing that seasonal acclimatisation leads to a greater reduction in body heat storage in older adults [40]. Increased sweating and the onset of sweating at a lower core temperature showcase a peripheral and core adaptation that results in greater cooling when exercising in the heat [8]. Improvements were seen in heart rate [25–27,29,31], mean arterial pressure [10,26], thermal comfort [29], thermal sensation [28,35] and rate of perceived exertion during exercise [35]. The findings from this review therefore suggest that HA may provide protective effects against heat exposure in an elderly population, but further investigation as to the magnitude of change attained from HA in older people is needed.

All participants completed the HA protocols with no adverse events. However, dropouts were reported in three studies [24,28,30]. Further, current HA protocols require specialised equipment and often do not cater for individuals unable to exercise. However, with some alterations to protocols, STHA could be utilised as a form of pre-emptive protection against hotter climates or adverse heat events. This could take the form of passive HWI that may be a pragmatic solution, but evidence regarding this approach is currently limited.

Waldock *et al*. [10] reported that if the exercise load became too difficult then it was reduced to enable the continuation of the protocol. Despite the decrease in workload, Waldock *et al*. [35] reported a reduction in T_re_ during the post-test simulated activities of daily living protocol and improved performance in a 6-minute walk test. Our systematic review therefore suggests HA protocols should include a modifiable exercise intensity for individuals unable to sustain target workload in the heat. A recent study suggests exercise in a thermoneutral environment followed by HWI and sauna use after exercise was effective at lowering core temperature, skin temperature and HR in younger participants, despite not showing an improvement in performance [41]. Therefore, we propose relative thermoneutral exercise followed by heat exposure may overcome difficulties of elderly populations exercising in the heat but this idea requires more investigation.

This review identified a number of key considerations when designing a STHA protocol for an older population. It was noted that the elderly participants had difficulty performing and the maintaining workload during HA from four of the studies [24,28,30,35]. In addition, this may have contributed to the negligible effects observed by Daanen and Herweijer [13]. This would suggest that a passive approach to HA would be more desirable for an older population but not obligatory. Participants were better equipped for the stresses imposed by STHA if they have a higher $\dot{\text{V}}{\text{O}}_{2\text{max}}$ [25,26]. However, this does not mean that those with a lower $\dot{\text{V}}{\text{O}}_{2\text{max}}$ cannot undertake the process. In fact, they stand to gain adaptations from a lower thermal stimulus [10]. A recent study by Ravanelli *et al*. [42] showed that participants with high and low $\dot{\text{V}}{\text{O}}_{2\text{max}}$ values showed greater sweat responses and lower core temperatures following an eight-week training programme. These findings suggests that training-induced thermoregulatory adaptations, not $\dot{\text{V}}{\text{O}}_{2\text{max}}$ alone, mediate the effects of heat stress. Therefore, active older individuals who participate in some form of aerobic training but not necessarily those with the highest $\dot{\text{V}}{\text{O}}_{2\text{max}}$ values, could perform better under heat stress than non-active individuals. Therefore, encouraging older populations to be more active is an important message as a counter measure against heat waves. If this is not an option, as may be the case with vulnerable individuals, both trained and untrained older men in their sixth decade have been shown to have a similar capacity for HA as younger men, as long as they are performing the same relative exercise intensity [29], however this could be superseded by the use of HWI.

A second consideration would be the length of the acclimation protocol and the consecutive nature of the protocol is performed over. STHA protocols should allow time for core temperature to reach 38.5ºC or +1.0°C from baseline. Nine of the twelve studies from this review performed consecutive day HA. In a recent review, Tyler *et al*. [43] stated that acclimation that is performed over consecutive days is more preferable than non-consecutive protocols however, ten days of consecutive HA conferred the same benefits as ten days of HA performed over a 27-day period (every third day) [44]. Given that heatwaves can be forecast up to twelve days in advance, this short window means time is of the essence and therefore consecutive HA should be utilised. However, undergoing HA on consecutive days is not always attainable. In these instances, undertaking some form of HA is more beneficial than doing nothing and this approach is perhaps more applicable to the general public e.g., before a heatwave. This approach is supported by Enander [45] who suggested that familiarity to a thermal environmental stress breeds competence when undertaking tasks in a hot environment. The protocol should be individualised to fulfil a specific purpose, allowing for adequate length of acclimation to gain physiological benefits.

Thirdly, three studies from this review demonstrated that older men have been shown to have a slower sweat response, as well as a blunted thirst response when compared to younger men [25,27,34]. This coupled with older populations having a decreased ability to recover from dehydration, as shown by Takamata *et al*. [33]. This could prove to be a problematic combination when undertaking STHA or experiencing hotter climates. To mitigate these issues, future STHA designs for older populations should include *ad lib* hydration and implement a minimum consumption and encouragement to drink more water. In addition, to a blunted psychological thirst response, Waldock *et al*. [10] identified a potential barrier for implementing heat preparation strategies in an ageing population. They found that when completing exercise tasks that equate to daily living, elderly people have decreased perceptual awareness, despite an elevation in markers of thermal strain. This suggests that elderly individuals may be less likely to implement a behavioural change in a thermally challenging environment. A more practical approach that can be carried out in a safe environment, that involves low intensity or no exercise component could be a feasible solution for heatwave protection which could be HWI however further research is required to understand if HWI could provide an acceptable, low intensity environment for heat acclimation.

There are still unanswered questions in the field of STHA and more research is required to fully explore the effectiveness and practical implications of HWI. Greenfield *et al*. [46] found that HWI resulted in substantial thermal strain and ‘partial’ heat acclimation for healthy participants. Only ‘partial’ acclimation could be attributed to the short exposure time which included three sessions of forty minutes in 40ºC, eliciting an average of only twenty minutes of core temperature ≥38.5ºC. An increased time spent in the water could stimulate a more substantial acclimation response. Zurawlew *et al*. [47] found that six-days of 40-minute running bouts at 60% $\dot{\text{V}}{\text{O}}_{2\text{max}}$ followed by post-exercise HWI into 40ºC evoked adaptions in non-acclimated males, more than exercise followed by thermoneutral water immersion. In addition, the adaptations were observed in trained and untrained participants and lasted up to two-weeks post-acclimation in similar studies [22,48]. Water transfers heat at a greater rate than air [49] and therefore could be used to rapidly induce heat acclimation. HWI could offer a suitable alternative to traditional HA methods, especially for populations that may have difficulty maintaining a workload and could also provide a mimetic to facilitate moving towards regular exercise.

Waldock *et al*. [10] was the only study in the review to explore the relatively novel concept of post-exercise HWI, as an acclimation method in an older population. Waldock demonstrated that post-exercise HWI could be utilised as an effective method of HA for an elderly population and postulated that it could be used to mitigate against heat-related illness caused by heatwaves. While this method utilised an exercise component, given the sedentary lifestyle of the elderly, future research involving an untrained, elderly population should investigate the HWI as a standalone intervention, like the method of Greenfield *et al*. [46]. Not all individuals are able to exercise, research investigating completely passive HWI protocols requires attention. There are also numerous other concerns from a safety standpoint such as if this was adopted as a “home prevention” programme then the need for regulated monitoring of core temperature and the condition of the person undertaking the protocol. In addition, transient hypotension caused by the transition of moving from seated to standing could increase the potential for falls in an older population. Further refinement of the concept and greater investigations into this area are required.

The majority of the participants from this review were male. This is important as hormone imbalances during menstruation impact female core temperature in younger population [6]. However, this would not be the case with post-menopausal women of an older age but information on this population is limited. Caution should therefore be made when generalising these findings to females. Further, all participants were ‘healthy’ and further research into the safety of HA in vulnerable populations is required.

## Conclusion

The twelve studies examined suggest that HA is feasible and efficacious an older population. Current HA protocols require specialised equipment and do not cater for individuals unable to exercise. Further thermoregulatory research should aim to include more female participants to explore differences in effectiveness of HA pre- and post- menopause. There remains very limited data on HWI and how it compares to other HA methods. However, HWI could serve as a viable and practical form of HA and remove some of the issues posed by traditional STHA protocols highlighted in this review. Given that HWI does not require specialist equipment, such as an environmental chamber, HWI could be a cost effective and time saving method of HA for general use, as well as used with populations who struggle with the exercise component of traditional HA methods.

## Supporting information

S1 Checklist PRISMA 2009 checklist. (DOCX)

## Abbreviations

$\dot{\text{V}}{\text{O}}_{2\text{max}}$: Maximum oxygen uptake [L^.^min^-1^or mL^.^kg^-1.^min^-1^]
$\dot{Q}$: Cardiac output [L^.^min^-1^]
${\bar{T}}_{sk}$: Mean skin temperature [°C]
$\dot{\text{V}}{\text{O}}_{2}$: Oxygen consumption [L^.^min^-1^or mL^.^kg^-1.^min^-1^]
${\bar{T}}_{b}$: Mean body temperature [°C]
$\dot{\text{V}}{\text{O}}_{2\text{peak}}$: Peak oxygen uptake [L^.^min^-1^or mL^.^kg^-1.^min^-1^]
HA: Heat acclimation
HST: Heat stress test
HWI: Hot water immersion
PV: Plasma volume
RH: Relative humidity [%]
STHA: Short-term heat acclimation
*T*_*re*_: Rectal temperature [°C]
